# Recapitulating the tumour microenvironment: advancing personalised radiation therapy through organoid technology

**DOI:** 10.1186/s13046-026-03655-0

**Published:** 2026-02-27

**Authors:** Zhen Lan, Jingyuan Liu, Lingling Li, Yuanyuan Yang, Xiaohui Zhu, Qiming Wang, Zhenqiang Sun, Yang Liu

**Affiliations:** 1 Department of Radiotherapy, Affiliated Cancer Hospital of Zhengzhou University, Henan Cancer Hospital, Zhengzhou, 450008 China; 2 Department of Internal Medicine, Affiliated Cancer Hospital of Zhengzhou University, Henan Cancer Hospital, Zhengzhou, 450008 China; 3 Department of Colorectal Surgery, The First Affiliated Hospital of Zhengzhou University, Zhengzhou, 450008 China

**Keywords:** Organoid, Tumor microenvironment, Radiotherapy, Heterogeneity, Hypoxia

## Abstract

The efficacy and resistance of tumor radiotherapy are profoundly influenced by the complex tumor microenvironment (TME). However, traditional preclinical models have limitations in accurately simulating the heterogeneity and dynamic interactions of the human TME, hindering the development of TME-targeted radiosensitization strategies. Organoid technology, as a revolutionary three-dimensional in vitro model, faithfully recapitulates the histological architecture, cellular diversity, and genetic characteristics of primary tumors. This provides a powerful platform for investigating the impact of the TME on radiotherapy response within a system that highly mimics physiological/pathological conditions.

This review systematically elaborates on how organoid technology guides tumor radiotherapy by modeling key components of the TME. We begin by outlining the features of organoid technology and its advantages in recapitulating TME heterogeneity. Subsequently, we delve into the latest applications of organoid models in deciphering how key factors within the TME—such as cell-cell interactions, immune cell functions, stromal components, and hypoxic conditions—influence radiotherapy response. Furthermore, this article summarizes the progress in using organoid technology to study radiosensitivity across different tumor types and highlights its great potential, particularly when integrated with multi-omics technologies, for uncovering mechanisms of radiotherapy resistance.

Based on this research, we emphasize the clinical translation prospects of organoid technology as a functional platform for guiding personalized radiotherapy strategies, including dose screening and combination therapy. Finally, we objectively discuss the current technical challenges faced by these models, including standardization, vascularization, immunocompetence, and clinical integration, and offer perspectives on future optimization directions and their broad clinical application potential. In conclusion, organoid technology holds promise for reshaping the research paradigm of tumor radiotherapy and advancing the era of individualized precision radiotherapy.

## Introduction

 Radiation therapy stands as one of the cornerstones of cancer treatment, with over half of all cancer patients requiring radiotherapy during their disease course. Its principle relies on utilizing ionizing radiation to induce irreversible DNA damage in tumor cells, thereby activating cell death programs. However, clinical efficacy is often compromised by intrinsic or therapy-induced radioresistance, which remains a major cause of local recurrence and distant metastasis. Accumulating evidence indicates that tumor radiosensitivity is not solely determined by the genetic characteristics of the tumor cells themselves, but is, to a great extent, precisely regulated by the complex ecosystem in which they survive – the Tumor Microenvironment (TME) [1, 2]. The TME is a dynamic functional unit composed of tumor cells, immune cells, cancer-associated fibroblasts, vascular endothelial cells, the extracellular matrix, and various signaling molecules [3, 4]. Within this unit, the functional status of immune components, the physical and biochemical support from stromal elements, and the prevalent hypoxic conditions have all been demonstrated as key factors influencing the radiotherapeutic response.

The successful implementation of precision radiotherapy urgently requires preclinical models that can faithfully mimic the complexity and heterogeneity of the in vivo human TME. However, development in this field has long been constrained by the inherent limitations of existing models. Traditional two-dimensional (2D) cell culture systems fail to recapitulate three-dimensional (3D) cell-cell and cell-matrix interactions and are prone to losing the heterogeneity of the parental tumor after multiple passages [5]. Patient-derived xenograft (PDX) models, while better preserving the tumor’s histological characteristics and heterogeneity, suffer from long modeling cycles, high costs, and an inability to fully recapitulate the human-specific immune microenvironment due to the prevalent use of immunodeficient mice, greatly limiting their application in research areas such as radioimmunotherapy [6, 7]. Therefore, the development of a novel in vitro model capable of highly simulating patient-specific TME characteristics is of paramount importance for deeply elucidating the mechanisms of radioresistance and developing novel radiosensitization strategies [8, 9].

In recent years, the emergence of organoid technology has brought a revolutionary breakthrough to this predicament. Organoids are three-dimensional (3D) cell clusters that self-organize from adult stem cells, embryonic stem cells, or induced pluripotent stem cells under in vitro 3D culture conditions, capable of highly simulating the tissue structure, cellular diversity, and key physiological functions of the source organ or tumor [10]. Particularly noteworthy, patient-derived tumor organoids (PDOs) can not only retain the genomic features, histopathological morphology, and drug sensitivity of the parental tumor but also reconstruct key components of its unique TME, including intrinsic cell-cell interactions, and partially, immune cells and stromal cells. This unprecedented biomimetic capability establishes tumor organoids as a powerful platform for systematically dissecting the specific mechanistic roles of various components within the TME (such as immune cells, cancer-associated fibroblasts, and hypoxic conditions) in regulating the radiotherapeutic response under highly controllable in vitro conditions [11, 12]. Furthermore, the establishment of organoid biobanks and their integration with high-throughput screening and multi-omics analysis technologies further solidify their immense potential in predicting personalized radiotherapy regimens [13–16].

This review aims to systematically elaborate on how organoid technology can guide and optimize tumor radiotherapy by modeling the TME. The article will begin by outlining the key characteristics of organoid technology and its unique advantages in simulating TME heterogeneity [17]. Subsequently, it will delve in a stratified manner into the latest research advances utilizing organoid models to reveal the impact of cellular crosstalk, immune components, stromal constituents, and hypoxic conditions within the TME on radiotherapy efficacy. It will also summarize the application of these models in studying radiosensitivity across different tumor types [10, 18]. Furthermore, we will discuss the value of integrating organoid technology with cutting-edge approaches such as multi-omics and microfluidic chips in addressing the challenge of radioresistance, and will explore its potential in advancing the clinical translation of personalized radiotherapy [7, 19, 20]. Finally, we will objectively evaluate the current technical challenges and limitations of organoid models and provide an outlook on their future directions [21]. Through this review, we seek to highlight the pivotal bridging role of organoid models between basic radiobiological research and clinical radiotherapy practice, thereby offering novel theoretical foundations and practical strategies to ultimately overcome radioresistance and achieve precision radiotherapy.

### Characteristics of organoid technology

Organoid technology, as a revolutionary three-dimensional in vitro model, has demonstrated remarkable progress in cancer research in recent years. Its core advantage lies in the ability to highly simulate the complex microstructure, cellular heterogeneity, and pathophysiological characteristics of the source tissue, thereby providing a powerful platform for precision medicine and translational research [20, 22]. By analyzing multiple studies, we can systematically delineate the core features and intrinsic logic of this technology [23].

The principal strength of organoid technology is its capacity to highly recapitulate tumor heterogeneity and tissue architecture. For instance, in glioblastoma (GBM) models, researchers have successfully generated glioblastoma organoids (GBOs) by directly culturing fresh tumor tissue fragments in a completely defined, serum-free medium, achieving a high success rate of 91.4%. This method, which avoids single-cell dissociation, not only prevents clonal selection induced by exogenous growth factors but also effectively preserves cell-cell interactions, local tissue structures (such as vessel-like structures and hypoxia gradients), and key molecular features (e.g., EGFRvIII mutation) of the parental tumor, thereby faithfully reproducing intra- and inter-tumoral heterogeneity [24]. Similarly, patient-derived organoids (PDOs) from colorectal cancer (CRC), esophageal adenocarcinoma (EAC), and triple-negative breast cancer (TNBC) have been confirmed to stably maintain the histological architecture and gene expression profiles of the original tumors, offering highly reliable in vitro models for studying tumor biology [25–27].

Building on its success in mimicking tumor complexity, another standout feature of organoid technology is its exceptional stability in long-term culture and experimental tractability. This characteristic enables the establishment of renewable living organoid biobanks, supporting long-term mechanistic investigations and reproducible high-throughput screening. For example, in intestinal stem cell research, organoid models allow for the stable isolation and culture of organoids from wild-type and gene-knockout (e.g., Ddx58-/-) mice, facilitating the precise assessment of specific gene functions (e.g., RIG-I) in intestinal stem cell regeneration post-irradiation within a controlled in vitro environment, free from the complexities of in vivo microbial and immune system interference [28]. Furthermore, the tractability of this technology is evidenced by the ease of genetic manipulation (e.g., using lentivirus for gene knockdown or overexpression) and its compatibility with various matrix materials (e.g., Matrigel), providing considerable flexibility for functional studies [29, 30].

Leveraging this high biomimicry and tractability, organoid technology demonstrates broad functional applicability, particularly in therapeutic response assessment and combination therapy development. These models have been extensively used to evaluate responses to standard therapies (e.g., radiotherapy, temozolomide chemotherapy), targeted therapies (e.g., EGFR inhibitors, PARP inhibitors), and even emerging immunotherapies (e.g., CAR-T). Studies have shown that GBO models can effectively simulate the efficacy of temozolomide combined with radiotherapy and the cytotoxic effects of CAR-T cells. More importantly, organoids serve as efficient screening platforms for combination strategies [24]. For instance, studies in CRC and EAC models have demonstrated that the ANO1 channel inhibitor and the ESRRA inhibitor significantly enhance tumor radiosensitivity, respectively, offering novel candidate strategies to overcome radioresistance [31, 32].

Notably, the translational value of organoid technology is substantially enhanced through its close integration and mutual validation with in vivo models, forming a complete research cycle from in vitro discovery to in vivo verification. Organoids not only function as standalone in vitro models but also, when derived into patient-derived organoid xenograft (PDOX) models, greatly improve the predictive reliability of preclinical data [17, 33]. For example, transplanting glioblastoma organoids into the brains of immunodeficient mice recapitulates their invasive growth characteristics, while in vivo experiments with colorectal cancer organoids visually demonstrate the tumor-suppressive effects of combining an ANO1 inhibitor with radiotherapy [31]. This synergistic validation between in vitro and in vivo models significantly accelerates the translation of basic mechanistic discoveries into potential clinical treatment strategies.

In summary, organoid technology, characterized by its high physiological relevance, stable cultivability, diverse functional applications, and effective complementarity with in vivo models, has firmly established itself as a critical bridge connecting basic tumor biology with clinical practice [34]. With ongoing technological optimization and standardization, organoid models are poised to play an indispensable core function in unraveling tumor mechanisms, accelerating drug development, and ultimately realizing truly personalized cancer therapy [35].

### Organoid technology simulates the heterogeneity of the tumour microenvironment

The profound heterogeneity of the tumor microenvironment (TME) is a central driver of malignant progression and therapy resistance. Comprehensively deconvoluting this complexity necessitates preclinical models that faithfully mimic the in vivo patient situation [10, 20]. Recent breakthroughs in organoid technology have introduced a revolutionary tool for this field. By systematically reconstructing the multi-dimensional heterogeneity of the TME within three-dimensional (3D) culture systems, it provides an unprecedented platform for uncovering its biological mechanisms under controlled conditions [36].

A core advantage of organoid models lies in their ability to achieve high-fidelity recapitulation of the TME’s complex cellular composition. Studies confirm that meningioma organoids derived from patient tissues not only retain tumor cells but also stably maintain the coexistence of key immune cells such as vascular endothelial cells, CD68⁺ macrophages, and CD3⁺ T cells; single-cell sequencing further reveals the presence of tumor cell subpopulations like SULT1E1⁺ cells [37]. Similarly, glioblastoma organoids (GBOs) also fully reconstitute the tumor ecosystem, including microvascular endothelial cells and microglia/macrophages, while preserving the diverse cell types of the original tumor, such as neural progenitor cells (e.g., SOX2+, NESTIN+), differentiated glial cells (e.g., GFAP+, S100B+), and immune cells (e.g., macrophages/microglia and T cells) [38]. Their immunohistochemical profiles show distribution patterns akin to the parental tumor [24]. This successful capture of the primary tumor’s cellular diversity establishes a solid foundation for in-depth in vitro investigation of dynamic interactions between tumor, stromal, and immune cells.

Beyond simulating cellular composition, the 3D growth characteristics of organoids enable the recreation of the spatial architectural heterogeneity of tumor tissues. For instance, colorectal cancer organoids can spontaneously form gland-like structures highly reminiscent of the primary tumor when embedded in appropriate extracellular matrices [39]. Furthermore, by orthotopically transplanting rectal cancer organoids into the corresponding mucosa of immunocompetent mice, studies have successfully replicated local invasion features and spatial distribution differences of internal signaling pathways (e.g., β-catenin and p-ERK) [40]. This simulation of spatial architecture is crucial as it provides a physiologically relevant context for studying cell polarity, intercellular communication, and regionalized signal transduction.

Building on the replication of cellular and structural layers, organoid technology demonstrates high fidelity in preserving tumor genetic heterogeneity. Multiple research teams have confirmed that colorectal cancer organoids stably maintain the mutational spectra of key driver genes like APC, TP53, and KRAS, along with the original subclonal architecture [41]. Head and neck cancer organoids also carry TP53 and NOTCH1 mutation profiles highly consistent with their parental tumors [42]. This genetic fidelity is not static but directly linked to functional output. For example, distinct subpopulations with different RAS-MAPK pathway mutations within rectal cancer organoids exhibit differential sensitivity to radiotherapy. Similarly, in head and neck cancer models, the efficacy of PRMT5 inhibitors is strictly dependent on the CDKN2A deletion status [21, 43]. These findings highlight the unique value of organoid models in connecting genotype to phenotype and elucidating the functional consequences of genetic heterogeneity.

Organoid models also exhibit a remarkable ability to accurately mimic clinical heterogeneity at the level of functional response. Across different cancer types, organoids precisely recapitulate the therapeutic response variations observed clinically: in triple-negative breast cancer organoids, cell subpopulations with differing NRP2 expression show significantly different radiosensitivity [44]. Meanwhile, hypoxic regions that spontaneously form during the growth of nasopharyngeal carcinoma organoids recreate the phenomenon of radiotherapy resistance mediated by HIF-1α pathway activation, with quantitative analysis revealing distinct radiobiological parameters compared to normoxic areas [45]. By combining live-cell imaging with single-cell analysis techniques, researchers can dynamically track metabolic heterogeneity during treatment or map the evolutionary trajectory of subclones within organoids [46], thereby systematically revealing the dynamic formation mechanisms of TME heterogeneity at a functional level [47].

Recent advancements in organoid models are also evident in the reconstruction of immune microenvironment heterogeneity. Improved co-culture systems, for instance in rectal cancer organoid models, can recreate immunosuppressive changes post-radiotherapy, such as M2 macrophage polarization and regulatory T cell infiltration. In-depth mechanistic studies further reveal that cancer cells can induce senescence in cancer-associated fibroblasts via IL-1α secretion, subsequently remodeling the stroma and excluding cytotoxic T cells, thereby delineating a complete heterogeneity regulation pathway from epithelial cells to the immune microenvironment [48].

In summary, organoid technology systematically and coherently reconstructs the panoramic heterogeneity of the TME through the layered simulation of cellular makeup, spatial architecture, genetic background, functional response, and immune microenvironment. This highly biomimetic platform not only provides unprecedented insights for basic research but also opens new avenues for the screening and prediction of individualized therapies. With the continuous refinement and integration of culture systems, organoid models are poised to play an increasingly pivotal role in the future development of precision oncology.

### Organoid technology simulates the effects of intercellular interactions in the tumour microenvironment on tumour radiotherapy

Organoid technology, utilizing three-dimensional (3D) culture systems to reconstruct the complex architecture of patient tumor tissues in vitro, provides a unique platform for studying the dynamic regulation of cell-cell interactions within the tumor microenvironment (TME) [7]. This technology maintains the spatial distribution and functional connections among various components such as tumor epithelial cells, stem cells, and stromal cells, thereby simulating critical biological processes including direct cell contact, paracrine signaling, and metabolic cooperation [22, 49]. Drawing upon multiple studies, this section systematically elaborates how organoid technology progressively reveals the multi-dimensional regulatory mechanisms of cell-cell interactions in radiotherapy response, from structural to functional levels.

At the structural level, organoid technology successfully models the impact of direct cell-cell contact on radiosensitivity. Research demonstrates that patient-derived organoids (PDOs) from rectal cancer retain the native spatial conformation of tumor and stromal cells in 3D culture, preserving direct interactions mediated by membrane surface molecules [30]. This structural integrity is crucial for radiotherapy response: upon irradiation, cell-contact relationships influence DNA homologous recombination repair efficiency by regulating the formation of the ATR-scaRNA2 complex, and scaRNA2 knockdown experiments confirm that this interaction significantly enhances radiosensitivity [30]. In intestinal organoids, radiation-induced apoptosis of Lgr5 + stem cells disrupts their supportive interaction with Paneth cells, further underscoring the fundamental role of direct cell contact in maintaining stem cell niche homeostasis and radioresistance [50]. The coexisting heterogeneity of cyst-like and crypt-like structures in colorectal cancer organoids also indicates that cell-cell contact and spatial organization differences are closely linked to radiotherapy response [51]. Furthermore, glioblastoma organoids (GBOs), cultured without single-cell dissociation, preserve the native cellular architecture and maintain direct contact between tumor cells and TME components (e.g., vascular endothelial cells, immune cells). Such interactions, modulated by hypoxia gradients (e.g., pimonidazole staining revealing core hypoxia), regulate cell proliferation and survival, thereby influencing radiosensitivity [24]. These studies structurally validate the advantage of organoids in simulating physical contacts.

**Fig. 1:**
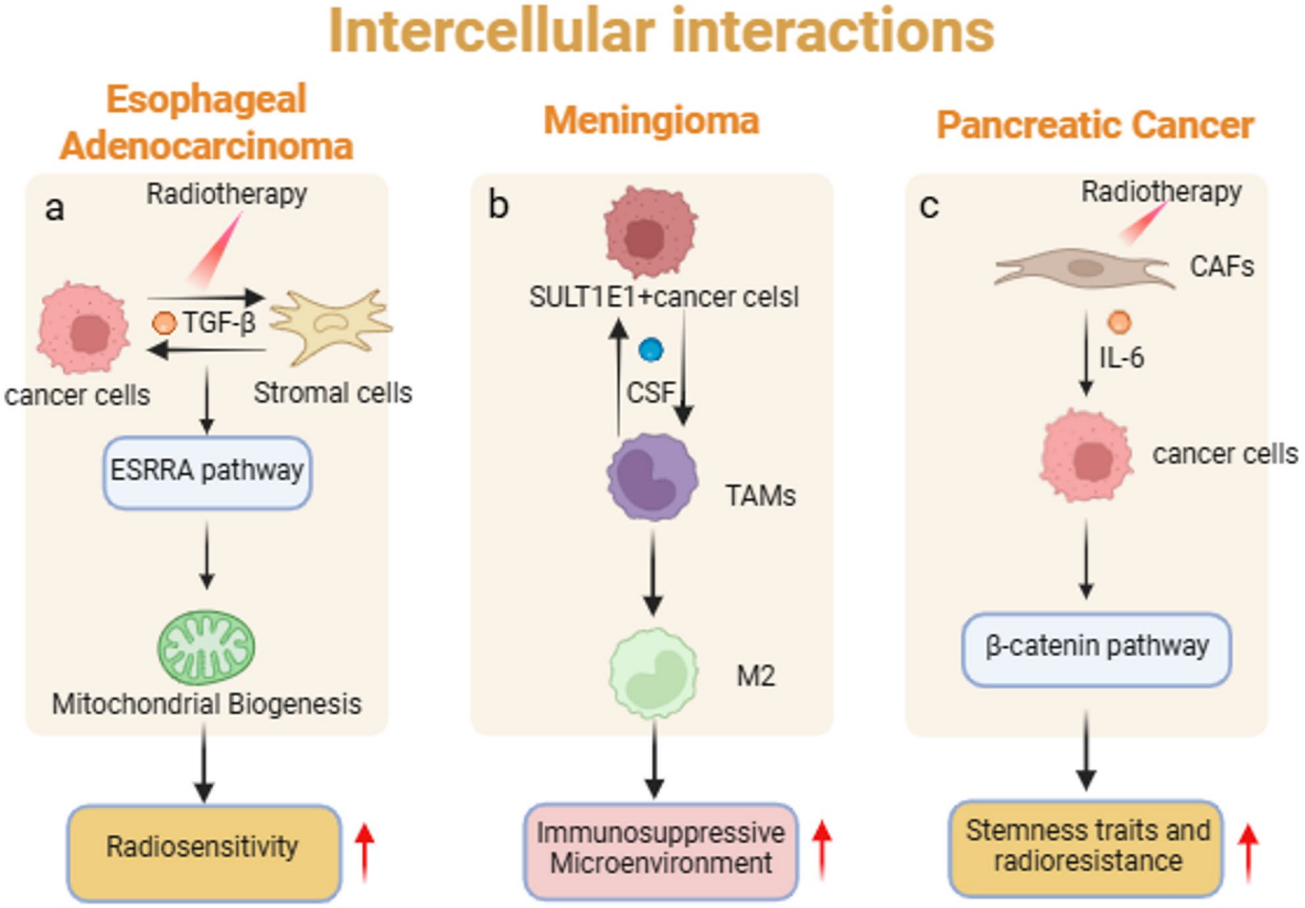
Intercellular interactions in the tumour microenvironment. **a** The paracrine crosstalk between tumor cells and stromal cells, mediated by factors such as TGF-β. **b** CSF signaling-mediated crosstalk between SULT1E1 + tumor cells and macrophages. **c** Radiation-induced cytokine secretion (such as IL-6) from CAFs activates the β-catenin pathway in tumor cells

Building upon structural simulation, organoid technology further recapitulates the dynamic regulation of radiotherapy response by paracrine signaling networks within the microenvironment. Studies on esophageal adenocarcinoma organoids show that radiation activates the ESRRA pathway and promotes mitochondrial biogenesis, a process involving paracrine crosstalk between tumor and stromal cells via factors like TGF-β [32] (Fig. 1a).Research on head and neck cancer organoids indicates that HPV-positive and HPV-negative models, differing in TP53 mutation status, modulate intercellular communication through NF-κB and cell cycle-related pathways, respectively, consequently affecting radiosensitivity [52]. In triple-negative breast cancer organoids, NRP2-high tumor cells act as nitric oxide signaling hubs, alleviating radiation-induced oxidative stress in neighboring cells via paracrine mechanisms, while targeting NRP2 enhances radiosensitivity [44]. Notably, targeted intervention of paracrine axes (e.g., combining the AKR1C3 inhibitor MPA with radiotherapy) can induce ferroptosis via the AKR1C3-HSPA5-GPX4 signaling pathway, significantly improving radiotherapy efficacy [26]. These findings highlight the value of organoids in modeling soluble factor-mediated cell communication.

The strength of organoid models is also evident in their ability to replicate multicellular interaction networks. Meningioma organoids, retaining SSTR2a+ tumor cells, CD68 + macrophages, and CD3 + T cells, reveal that SULT1E1 + tumor subpopulations mediate crosstalk with macrophages via CSF signaling, driving their polarization towards an M2 phenotype and fostering an immunosuppressive microenvironment [37] (Fig. 1b). In co-culture models of pancreatic cancer organoids and cancer-associated fibroblasts (CAFs), radiation induces CAFs to secrete cytokines like IL-6, which subsequently activates the β-catenin pathway in tumor cells, enhancing their stemness and radioresistance [53] (Fig. 1c). Studies on salivary gland organoids also found that radiation induces cellular senescence and the senescence-associated secretory phenotype (SASP), influencing surrounding cell function via paracrine signals [54]. These models successfully simulate the integrated effects of stromal-tumor-immune tripartite interactions in radiotherapy response.

At the molecular mechanism level, organoids are widely used to dissect downstream signaling pathways triggered by cell-cell interactions. In meningioma organoid studies, combining a FOXM1 inhibitor with radiotherapy significantly suppressed organoid proliferation, suggesting that intercellular interactions influence radiotherapy response by regulating proliferation-related signaling networks [55]. Intestinal organoid research confirmed that radiation regulates stem cell fate decisions by activating the Notch pathway, with single-cell analysis showing specific enrichment of ligand-receptor pairs among different stem cell subpopulations [28]. In colorectal cancer organoids, PARP13 loss disrupts multicellular spheroid integrity and enhances radiosensitivity, an effect not prominent in 2D culture, underscoring the important regulatory role of the 3D microenvironment in signal transduction [39]. These studies elucidate, at the molecular level, how intercellular communication collectively determines radiotherapy efficacy.

Organoid technology has further elucidated the roles of metabolic cooperation and clonal dynamics in radiotherapy responses. In liver cancer organoids, Raman spectroscopy analysis revealed that combined treatment with radiotherapy and a ferroptosis inducer induces heterogeneous alterations in glutathione metabolism and fatty acid composition, suggesting metabolic collaboration among cells through antioxidant defense systems [56]. Intestinal organoid studies have demonstrated that radiation regulates ferroptosis via the STAT1-IRF1-ACSL4 pathway, highlighting the significance of intercellular interactions at the metabolic level [57]. Regarding clonal dynamics, single-cell whole-genome sequencing of patient-derived rectal cancer organoids showed that radiotherapy significantly alters subclonal composition, reflecting clonal evolution driven by intercellular competition for survival signals within the tumor microenvironment [47].

Despite significantly advancing our understanding of microenvironmental interactions, organoid technology still faces limitations in terms of component completeness [58]. The frequent loss of stromal cells in standard culture conditions may impair the comprehensive simulation of intercellular interaction networks. Consequently, current research is focusing on optimizing co-culture systems, such as by incorporating patient-derived immune cells or fibroblasts, to more accurately reconstitute the multicellular signaling networks present within the native tumor microenvironment [40, 59].

In summary, organoid technology, through its three-dimensional culture system, systematically uncovers the multidimensional regulatory mechanisms governed by intercellular interactions within the tumor microenvironment that determine the response to radiotherapy [60]. This platform not only deepens our comprehension of radioresistance mechanisms but also provides a systematic research tool for developing novel combination therapies that target microenvironmental crosstalk [17, 33]. With continued refinement of co-culture systems, organoid models are poised to play an increasingly pivotal role in translational research aimed at advancing precision radiotherapy [61].

### Organoid technology simulates the impact of immune components in the tumour microenvironment on tumour radiotherapy

Immune components within the tumor microenvironment (TME) significantly modulate the response to radiotherapy through a complex network of intercellular interactions. Organoid technology, as an emerging three-dimensional (3D) in vitro platform, provides a unique system for deconvoluting this intricate regulatory relationship. By integrating various immune cell types in co-culture with tumor cells, this technology can not only simulate the spatial distribution and direct contact between immune and tumor cells but also recapitulate critical biological processes such as immunomodulatory signaling and metabolic cooperation [10, 61–64].

In terms of model establishment, organoid technology has developed multiple strategies to mimic the tumor immune microenvironment. By optimizing culture conditions, researchers have successfully preserved key immune components—such as CD68⁺ macrophages and CD3⁺ T cells—within patient-derived meningioma organoids, thereby maximizing the fidelity to the individual’s original immune contexture [37]. Concurrently, active co-culture strategies have further expanded the applicability of organoids. For instance, in co-culture models of pancreatic cancer organoids and cancer-associated fibroblasts (CAFs), researchers have modeled the IL-1α signaling-mediated polarization of inflammatory CAFs and observed their regulatory effect on CD8⁺ T cell infiltration [48].

At the functional level, organoid platforms have successfully replicated key immune-mediated biological processes. Studies using glioblastoma organoids revealed that tumor-associated macrophages secrete immunosuppressive factors such as IL-10 and TGF-β, activating the NF-κB signaling pathway in tumor cells and consequently inducing radioresistance [38]. Moreover, paracrine signaling from immune cells—via cytokines including TNF, IL1B, and TGFB1—was shown to influence DNA damage repair and apoptotic pathways [24]. Complementing these findings, studies in immunocompetent rectal cancer organoids demonstrated that radiotherapy promotes M2-like macrophage polarization and regulatory T cell expansion, leading to suppression of antitumor immunity through immune checkpoint molecules such as PD-1/PD-L1 and CTLA-4 [40]. These insights provide important mechanistic underpinnings for understanding radiotherapy-induced immunosuppression.

Notably, organoid technology enables exploration of the complexity of cross-talk between multiple signaling pathways within the immune microenvironment. Research on esophageal adenocarcinoma organoids indicated that radiation-induced activation of the ESRRA pathway promotes mitochondrial biogenesis, a process potentially affecting immune cell function through mediators like TGF-β [32]. Further studies in head and neck cancer organoids suggested that TP53 mutation status modulates intercellular communication via the NF-κB signaling pathway, offering new perspectives on the role of signaling networks in radiotherapy-associated immune regulation [52].

At the metabolic level, organoid models provide a unique vantage point for investigating metabolic interactions between immune and tumor cells. Raman spectroscopic analysis in liver cancer organoids indicated that radiotherapy induces heterogeneous alterations in glutathione metabolism and fatty acid composition, implying potential metabolic competition between tumor and immune cells [56]. In triple-negative breast cancer organoids, NRP2-high tumor cells were found to modulate oxidative stress, potentially influencing immune cell function [44]. These findings deepen our understanding of immunometabolic regulation in the irradiated TME.

Despite notable progress, organoid technology still faces limitations regarding immune component stability [58]. The gradual loss of immune cells during passaging under standard culture conditions, as well as the general lack of a complete immune repertoire in many models, constrains long-term investigation [41]. To address these issues, researchers are employing various engineering strategies—such as generating “immunized organoids” and utilizing 3D bioprinting to simulate immune cell infiltration [27, 59].

In summary, organoid technology, through the establishment of immune cell co-culture systems, is progressively refining the simulation of the tumor immune microenvironment across multiple levels. As engineering techniques continue to advance, this platform holds great promise for elucidating the immune regulatory mechanisms of radiotherapy and developing novel combination strategies, ultimately facilitating the transition toward a more precise paradigm in cancer radiotherapy.

### Organoid technology simulates the effects of tumour microenvironment matrix components on tumour radiotherapy

Organoid technology, leveraging its unique three-dimensional (3D) culture system, provides a dynamic platform for dissecting the complex stromal components within the tumor microenvironment (TME). This technology first enables the reconstruction of the basic structure and function of the extracellular matrix (ECM)—utilizing matrices like Matrigel to mimic the biochemical composition of the native basement membrane, thereby providing tumor cells with crucial physical support and biochemical signaling cues. Studies demonstrate that in glioblastoma organoids, an ECM rich in collagen and fibronectin forms a physical barrier that significantly attenuates the cytotoxic effects of radiation on deep-seated cells, an effect that can be reversed by collagenase pretreatment [38]. Similarly, in colorectal cancer organoids, the Matrigel environment influences DNA damage repair efficiency by regulating the SENP5-H2AZ axis, revealing an active role of ECM components in the radiation response [65].

Building on this foundation, organoid technology overcomes the limitations of static ECM simulation by incorporating viable stromal cells, enabling dynamic analysis of tumor-stroma interactions. The integration of cancer-associated fibroblasts (CAFs) is particularly significant: in glioblastoma models, co-cultured CAFs enhance the DNA repair capacity of tumor cells via hypoxia-induced HIF-1α secretion [38]. Meanwhile, meningioma organoids successfully retain CD31⁺ vascular endothelial cells from the primary tumor, constructing a complex microenvironment that includes vascular networks [37]. These models demonstrate that CAFs not only mediate radioresistance through paracrine signals (e.g., IL-6, TGF-β) but also drive ECM remodeling, creating dual physical and biochemical barriers.

Furthermore, organoid models elucidate the molecular pathways through which stromal components regulate radiosensitivity. Interactions between the ECM and cell surface integrins activate intracellular survival signaling—in glioblastoma organoids, integrins αvβ3/β5 inhibit radiation-induced apoptosis via the PI3K-Akt pathway [38]. Liver cancer organoids derived from cirrhotic backgrounds show that high-stiffness ECM transmits mechanical signals through the integrin β1-FAK axis, impairing the DNA damage response [66]. Beyond mechanotransduction, organoids also recapitulate stroma-associated metabolic support functions: in intestinal organoids, the microbial metabolite indole-3-aldehyde mimics the protective effect of stromal-derived factors on epithelial cells via the AhR/IL-10 axis [67, 68]. In cerebral organoids, the microenvironment constituted by astrocytes influences glioma cell response to fractionated radiotherapy.

Organoid technology also exhibits the advantage of capturing spatiotemporal dynamics. Cardiac organoids post-irradiation show an upregulation of TGF-β and a progression of collagen deposition, modeling fibrosis. In small intestinal organoids, radiation injury triggers the WNT4-ROR2-β-catenin signaling axis [69], recapitulating the stroma-mediated epithelial repair mechanism [29]. These simulations of dynamic processes elevate stromal components from static structures to functional units actively participating in the radiotherapy response.

Although standard organoid models may lose primary stromal cells during passaging, this simplification underscores their scientific value: for instance, head and neck squamous cell carcinoma organoids, by comparing pure epithelial models with in vivo responses, indirectly confirm the critical role of CAFs in clinical radioresistance [58]. This “reductionist” strategy lays the groundwork for targeted mechanistic investigation.

In summary, organoid technology systematically reconstructs the multi-layered functions of stromal components within the TME, progressing from ECM simulation to cellular integration, and from signaling pathway dissection to the capture of dynamic processes. This platform not only deepens our understanding of stroma-mediated radioresistance mechanisms but also provides a translational pathway for developing personalized treatment strategies that target tumor-stroma interactions.

### Organoid technology simulates the effects of tumour hypoxia on tumour radiotherapy

The hypoxic tumor microenvironment is a key driver of radioresistance in solid tumors. Organoid technology, through its unique three-dimensional (3D) culture system, provides a highly biomimetic platform for simulating this complex pathophysiological scenario [7, 17, 33]. This technology not only recapitulates the physical basis of hypoxia but also systematically reveals its multi-layered biological effects, thereby advancing the in-depth understanding of hypoxia-mediated radioresistance mechanisms.

**Fig. 2:**
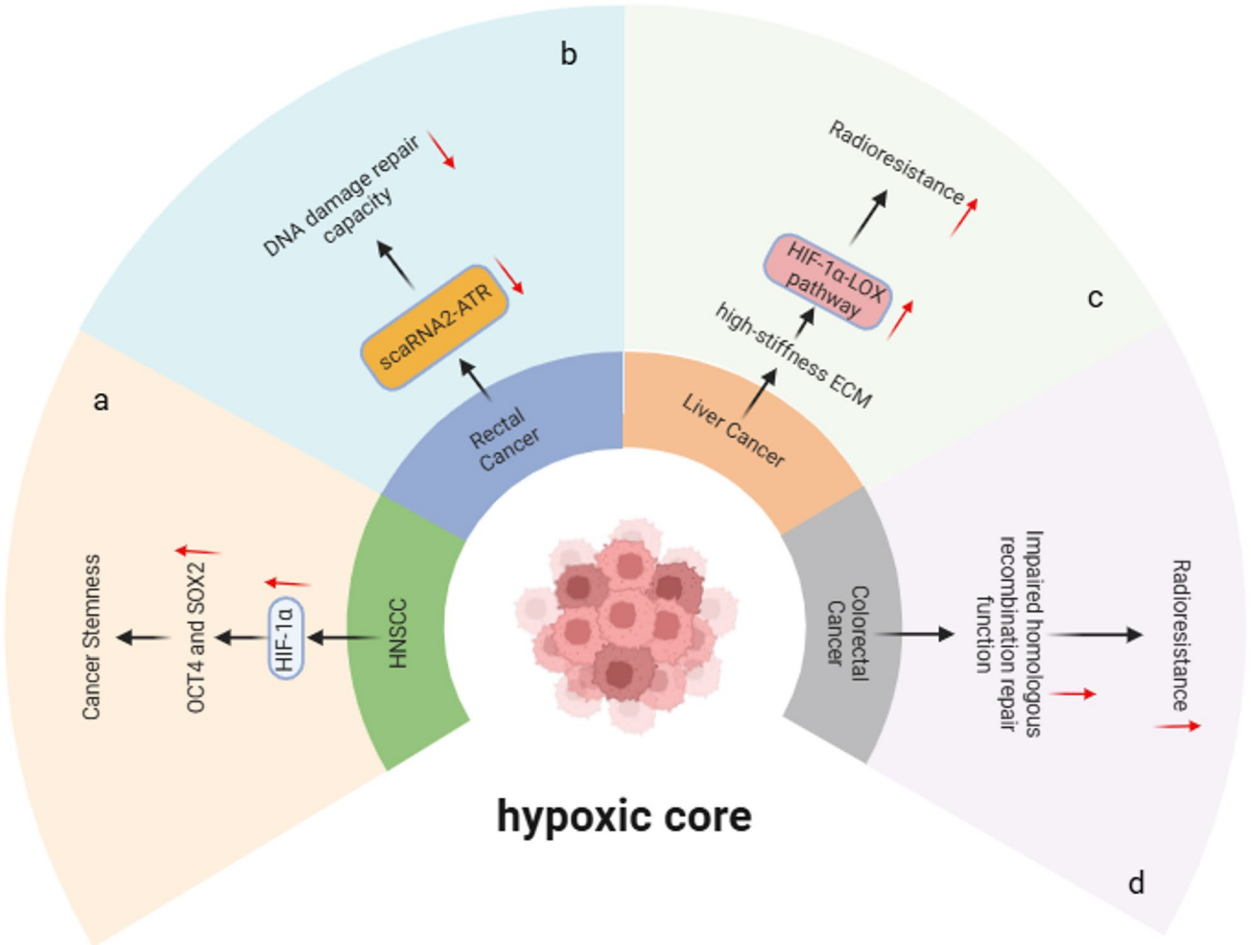
Simulated hypoxic microenvironment. **a** Hypoxia induces the upregulation of stemness genes (e.g., OCT4 and SOX2) via HIF-1α stabilization to maintain cancer stem cell properties. **b** Hypoxia-mediated regulation of DNA damage repair operates through the scaRNA2-ATR signaling pathway. **c** Hypoxia-induced activation of the HIF-1α-LOX pathway contributes to increased radioresistance. **d** Hypoxia-induced alterations in homologous recombination repair efficiency lead to modulated radiosensitivity

The 3D architecture of organoids spontaneously generates a gradient of decreasing oxygen partial pressure from the exterior to the interior, mimicking the spatial characteristics of hypoxia in solid tumors by limiting oxygen diffusion. For instance, the dense core regions of glioblastoma organoids naturally develop a hypoxic state (O₂ concentration < 0.1%) [24, 38], while esophageal adenocarcinoma organoids cultured in Matrigel also reproduce similar oxygen gradients [32]. This structural hypoxia provides not only a physical foundation for research but also directly triggers subsequent molecular responses. Building on this, organoid models successfully simulate the activation of key hypoxia-related signaling pathways. Studies show that esophageal adenocarcinoma organoids activate the ESRRA pathway under hypoxic conditions, promoting mitochondrial biogenesis and upregulating oxidative phosphorylation, thereby enhancing radioresistance [32]. Conversely, in head and neck squamous cell carcinoma organoids, hypoxia stabilizes HIF-1α, upregulating stemness genes like OCT4 and SOX2, and maintaining cancer stem cell properties [53] (Fig. 2a). The activation of these molecular events provides a mechanistic basis for further understanding the functional consequences of hypoxia.

Notably, hypoxia-triggered signaling pathway alterations are closely linked to cellular metabolic reprogramming. Organoid models can clearly reveal this connection. For example, in head and neck squamous cell carcinoma organoids, the hypoxic gradient formed by tight spheroid structures is significantly correlated with a glycolytic metabolic phenotype, highlighting the role of the “hypoxia-glycolysis” axis in driving radioresistance [70]. Similarly, in cardiac organoids, post-irradiation mitochondrial dysfunction and enhanced glycolysis mimic the Warburg effect observed in tumor cells. This metabolic remodeling affects not only energy supply but also indirectly influences treatment response by modulating cell fate decisions [69].

On the foundation of metabolic adaptation, organoid technology further uncovers the profound impact of hypoxia on genomic stability. Research on rectal cancer organoids indicates that the hypoxic microenvironment regulates DNA damage repair capacity via the scaRNA2-ATR signaling pathway [30, 41] (Fig. 2b). Similarly, studies on colorectal cancer organoids demonstrate that hypoxic conditions can alter radiosensitivity by affecting homologous recombination repair efficiency (Fig. 2d). These findings refine the understanding of hypoxia-mediated radioresistance from the perspective of the DNA damage response, offering new avenues for developing targeted strategies.

Beyond cell-intrinsic mechanisms, organoid models can capture the complex interplay between hypoxia and other microenvironmental factors. In liver cancer organoids modeling a cirrhotic background, high matrix stiffness compresses internal structures, exacerbating hypoxia and activating the HIF-1α-LOX pathway, thereby enhancing radioresistance [66] (Fig. 2c). Concurrently, macrophages and vascular structures retained in meningioma organoids may indirectly regulate the fate of cells in hypoxic regions through intercellular communication [37]. These interactions suggest that the functional output of the hypoxic niche is co-regulated by multiple factors.

To more accurately simulate the dynamic features of hypoxia, engineered organoid models have emerged. The CiGiP system, through its stacked design, not only simulates hypoxia but also reveals the impact of the post-radiotherapy reoxygenation process on cell fate [71]. In the future, integrating technologies like 3D bioprinting and microfluidics to construct perfusable vascular networks holds promise for reconstructing physiological oxygen gradients, further enhancing the predictive value of organoids in hypoxia research [21].

In summary, organoid technology, leveraging its 3D culture properties, systematically models the core features of the tumor hypoxic microenvironment—from physical structure and molecular responses to metabolic remodeling and microenvironmental crosstalk. This platform not only deepens the understanding of hypoxia-mediated radioresistance mechanisms but also provides a powerful tool for developing combination therapies targeting hypoxia. With the continuous advancement of engineering technologies, organoid models are poised to play an increasingly significant role in personalized radiotherapy research.

### The impact of organoid technology on different types of tumour radiotherapy

Organoid technology has emerged as a pivotal in vitro model platform in recent years. Its capacity to highly preserve the cellular heterogeneity, microenvironment features, and molecular phenotypes of primary tumors provides an unprecedented window for in-depth investigation into the response mechanisms of different tumor types to radiotherapy. By recapitulating the tissue architecture within three-dimensional (3D) culture systems, this technology not only overcomes the limitations of traditional two-dimensional (2D) models in simulating cell-cell interactions and signaling pathways but also, through the integration of multi-omics analysis, drug screening, and gene editing, systematically reveals the intrinsic principles governing radiosensitivity and resistance mechanisms (Table 1). This offers a solid experimental foundation for developing personalized radiotherapy strategies [7, 52, 72].

**Table 1:** The application of organoid technology in cancer

Cancer	ClinicalTrials.gov ID	objective	Start-Completion
Unresectable Perihilar Cholangiocarcinoma	NCT03307538	For patients with perihilar cholangiocarcinoma, surgery is the only treatment modality that can result in cure. the investigators designed a local feasibility trial with SBRT after chemotherapy in patients with unresectable perihilar cholangiocarcinoma in order to try to confirm the observed tolerability of adding SBRT to standard chemotherapy.	Study Start (Actual) : 2017-11-06 Study Completion (Estimated) : 2020-06-30
Breast cancer patients with brain metastases	NCT06468124	Patient-derived organoids (PDOs) generated from extracranial metastases obtained from brain resection or biopsy. The preliminary data collected will be used to evaluate the ability of PDOs to predict patient treatment response, and their radiosensitivity and chemotherapeutic sensitivity can be correlated with their survival outcomes.	Study Start (Actual) : 2024-07-01 Study Completion (Estimated) : 2025-07
Lung Cancer	NCT05092009	Lung (tumor) material obtained from biopsies or surgical resection material that is not needed by the pathologist for diagnosis (i.e. to stage the patient or to perform molecular diagnosis) will be collected and used for the generation of matched normal and lung cancer organoids and/or PDX.	Study Start (Actual) : 2022-10-05 Study Completion (Estimated) : 2028-04-01
Medulloblastoma	NCT06959979	Medulloblastoma (MB) is a rare but significant paediatric brain tumour. CDK4/6 inhibitors (palbociclib, ribociclib, abemaciclib) have been approved for breast cancer and show potential in other tumours, but their efficacy in MB remains unclear. Treatment resistance is a concern. This project aims to identify genetic markers of sensitivity to CDK4/6 inhibitors in MB to improve treatment and overcome resistance.	Study Start (Actual) : 2024-11-25 Study Completion (Estimated) : 2026-08-31
Pancreatic Cancer	NCT04931394	This study aims to investigate whether adjuvant chemotherapy regimens guided by organoid drug sensitivity testing can improve the prognosis of pancreatic cancer. Additionally, this study will evaluate the success rate of organoid establishment in fresh surgical specimens and explore the consistency between drug sensitivity test results and patient treatment responses.	Study Start (Actual) : 2021-06-01 Study Completion (Estimated) : 2025-05-31
Advanced Pancreatic Cancer	NCT04931381	The purpose of this study is to explore whether chemotherapy regimens guided by organoid drug sensitivity test can improve the outcomes of advanced pancreatic cancer. At the same time, this study will evaluate the successful stablishment rate of organoid from biopsy tissue, and explore the concordance between drug sensitivity test results and patients’ treatment response.	Study Start (Actual) : 2021-06-01 Study Completion (Estimated) : 2025-05-31
Cervical Cancer	NCT06786780	By constructing cervical cancer organoids, establishing a radiochemotherapy prediction model based on organoids, and exploring radiotherapy resistance-related molecules as new therapeutic targets for locally advanced cervical cancer, the feasibility and effectiveness are assessed. Utilizing the constructed model for molecular mechanism discussions and effective drug screening, new avenues for the treatment of cervical cancer patients with radiochemotherapy resistance are sought.	Study Start (Actual) : 2023-01-01 Study Completion (Estimated) : 2026-06-01
Wilms Tumor cancer	NCT04968990	To determine whether the application of proton beam radiotherapy to the lateral abdomen for conformal target volume reduction can promote normal growth of the lateral abdomen, compared with the contralateral untreated side and patients who did not receive radiotherapy. For patients with stage V (bilateral Wilms’ tumour) and specific surgical margins involved, proton beam radiotherapy (partial kidney proton beam radiotherapy) was administered to the involved kidney to assess whether it could maintain the same high control rate as traditional lateral abdominal/entire kidney irradiation fields.	Study Start (Actual) : 2021-08-19 Study Completion (Estimated) : 2036-12
Prostate Cancer	NCT07004582	For men with an aggressive form of prostate cancer, finding the right and effective treatment right away is challenging. Many of these men face a high risk of cancer recurrence: about half experience a relapse after surgery, and more than a third after undergoing radiation therapy. The aim of this project is to investigate which of these two techniques is most effective for testing and personalised treatment.	Study Start (Actual) : 2025-10-01 Study Completion (Estimated) : 2031-12-30
Glioblastoma (GM)	NCT04868396	The median overall survival for glioblastoma (GBM) patients is approximately 15 months. Standard treatment for GBM includes maximal surgical resection followed by radiotherapy and chemotherapy using temozolomide (TMZ). Regardless of the initial tumour response, tumour recurrence is inevitable. The aim of this study is to establish organoid cultures derived from primary patient-derived GM samples to investigate the mechanisms underlying the aggressive tumour growth and treatment resistance in both primary and recurrent GM.	Study Start (Actual) : 2021-04-10 Study Completion (Estimated) : 2025-09-01
non-small cell bronchopulmonary cancer (NSCLC)	NCT04826913	The primary objective of the study is to construct organoids from patients in order to select the best anticancer therapy for patients through screening devices. The secondary objective is to collect lymphocytes from patients’ blood to test the effectiveness of immune-mediated therapies (immunotherapy).	Study Start (Actual) : 2021-04 Study Completion (Estimated) : 2024-04
Esophageal Cancer	NCT03283527	The aim of the study was to create patient-derived organoid models for EC patients to predict the pathological response of tumours to nCRT in clinical practice. This will enable a more personalised approach to the treatment of locally advanced EC in the future.	Study Start (Actual) : 2017-12-01 Study Completion (Estimated) : 2023-12-31
inoperable or advanced solid tumors	NCT06077591	The aim of this study is to determine the clinical efficacy of NGS/PDO drug screening-guided treatment for patients with refractory/advanced solid tumours who are resistant to conventional chemotherapy. The in vitro response of PDO drugs will be correlated with the clinical response of these patients. The hypothesis is that WES and PDO drug screening can accurately identify candidate drugs that will reduce tumour size and benefit these patients.	Study Start (Actual) : 2024-10-18 Study Completion (Estimated) : 2028-02-01
Breast Cancer	NCT05464082	This is a prospective phase 2 study to use Functional Precision Oncology (FPO) to predict, prevent and treat early metastatic recurrence in subjects with HR-low/Her2 negative or triple negative breast cancer.	Study Start (Actual) : 2023-01-06 Study Completion (Estimated) : 2028-09-30
Rectal Cancer	NCT03577808	Patients with locally advanced rectal cancer will undergo biopsy prior to standard neoadjuvant chemoradiotherapy treatment. The sensitivity of radiotherapy and chemotherapy drugs will be tested in organoid models. The aim is to validate whether organoids can predict clinical outcomes in patients with locally advanced rectal cancer undergoing neoadjuvant chemoradiotherapy.	Study Start (Actual) : 2018-08-17 Study Completion (Estimated) : 2020-11-01
Rectal Cancer	NCT05352165	The aim was to randomly assign patients with locally advanced rectal cancer requiring neoadjuvant therapy prior to curative surgery into an organoid drug sensitivity group and a standard full neoadjuvant therapy group. The tumour pathological complete response rate (pCR), postoperative complication rate, postoperative tumour regression grade, postoperative recurrence rate, treatment tolerance rate, R0 resection rate, and sphincter preservation rate were observed and compared.	Study Start (Actual) : 2023-01-01 Study Completion (Estimated) : 2025-12-31
Rectal Cancer	NCT04842006	The aim of the study is to compare the results of a novel, precise method for selecting the right rectal cancer patients for the treatment they need with those of traditional treatment. The goal is to reduce overtreatment in those who are most unlikely to benefit from additional treatment. With the overall goal of reducing disease metastasis, patients with high-risk characteristics will be randomly assigned to one of two treatment strategies: early systemic control through chemotherapy, followed by ctDNA- and organoid-guided adjuvant therapy, or conventional treatment strategies.	Study Start (Actual) : 2021-12-20 Study Completion (Estimated) : 2031-12-31
Salivary Gland Carcinoma	NCT06047236	Aims of this study are analyses of tumor metabolome, tumor transcriptome and tumor proteome as well as of the immune infiltration, separated by histological entity. These data will subsequently be compared with the with the detailed immune status determined in the patient’s peripheral blood and saliva using machine learning techniques, among others, to create a biomarker cluster for salivary gland tumors.	Study Start (Actual) : 2024-01-08 Study Completion (Estimated) : 2029-09-30
Head and neck squamous cell carcinoma	NCT04261192	The aim of the study was to establish squamous cell organoids of the head and neck to assess their response to innovative therapies.	Study Start (Actual) : 2020-07-03 Study Completion (Estimated) : 2031-02
Glioma	NCT06156150	This study will be conducted using cell-based, organoid platform, and animal experiments to confirm the role and mechanisms of B7-H4 derived from macrophages in T-cell chemokine secretion and vaccine treatment resistance. Additionally, the produced DC vaccine will be used to assess the possibility of reversing vaccine resistance when intervening in the B7-H4 axis. Finally, a model to evaluate the clinical benefits of the vaccine will be established based on clinical trial data combined with B7-H4 expression and clinical pathological characteristics.	Study Start (Actual) : 2023-11-26 Study Completion (Estimated) : 2026-12-31
Breast Cancer	NCT05134779	The aim of the study is to establish a biobank of patient-derived tumour organoids (PDOs) from tumours obtained during surgery, either before or without neoadjuvant therapy (NAT), and at the time of recurrence/metastasis. The objective is to elucidate the complex interactions between genes, the cancer microenvironment, and the immune system to develop appropriate therapies.	Study Start (Actual) : 2022-01-12 Study Completion (Estimated) : 2028-12-31
Head and Neck Cancer	NCT05375266	The aim is to investigate the prognostic value of tumour-intrinsic and systemic immune biomarkers in newly diagnosed non-metastatic head and neck cancer. In addition, local immune processes in the tumour will be correlated with systemic immune status determined in peripheral blood to identify prognostic immune signatures. Furthermore, tumour organoids will be generated in vitro for functional bioanalysis. The main objective is to create a prognostic score determined by clusters based on tumour immunological criteria.	Study Start (Actual) : 2022-05-16 Study Completion (Estimated) : 2027-03-31
Rectal Cancer	NCT03874559	The primary objective of this study is to characterise the levels of exosomal biomarkers in patients with locally advanced rectal cancer undergoing neoadjuvant chemoradiotherapy. The exosomal expression rates before, during, and after chemoradiotherapy will be compared with the pathological response rates at APR or LAR. Researchers will also examine the functional roles of these exosomes in malignant colon organoids and colorectal cancer mouse models.	Study Start (Actual) : 2018-02-13 Study Completion (Estimated) : 2026-02-01
Soft Tissue Sarcoma	NCT02910895	Currently, systemic treatment for sarcomas includes older cytotoxic chemotherapy and newer targeted therapies, such as tyrosine kinase inhibitors. This study could form the basis for preclinical translational research on radiotherapy and various systemic treatments. Development of patient-derived xenografts (PDX) and soft tissue sarcoma (STS) 2D/3D cell culture platforms: a protocol for obtaining tumour material from STS patients.	Study Start (Actual) : 2017-09-09 Study Completion (Estimated) : 2024-09

**Fig. 3:**
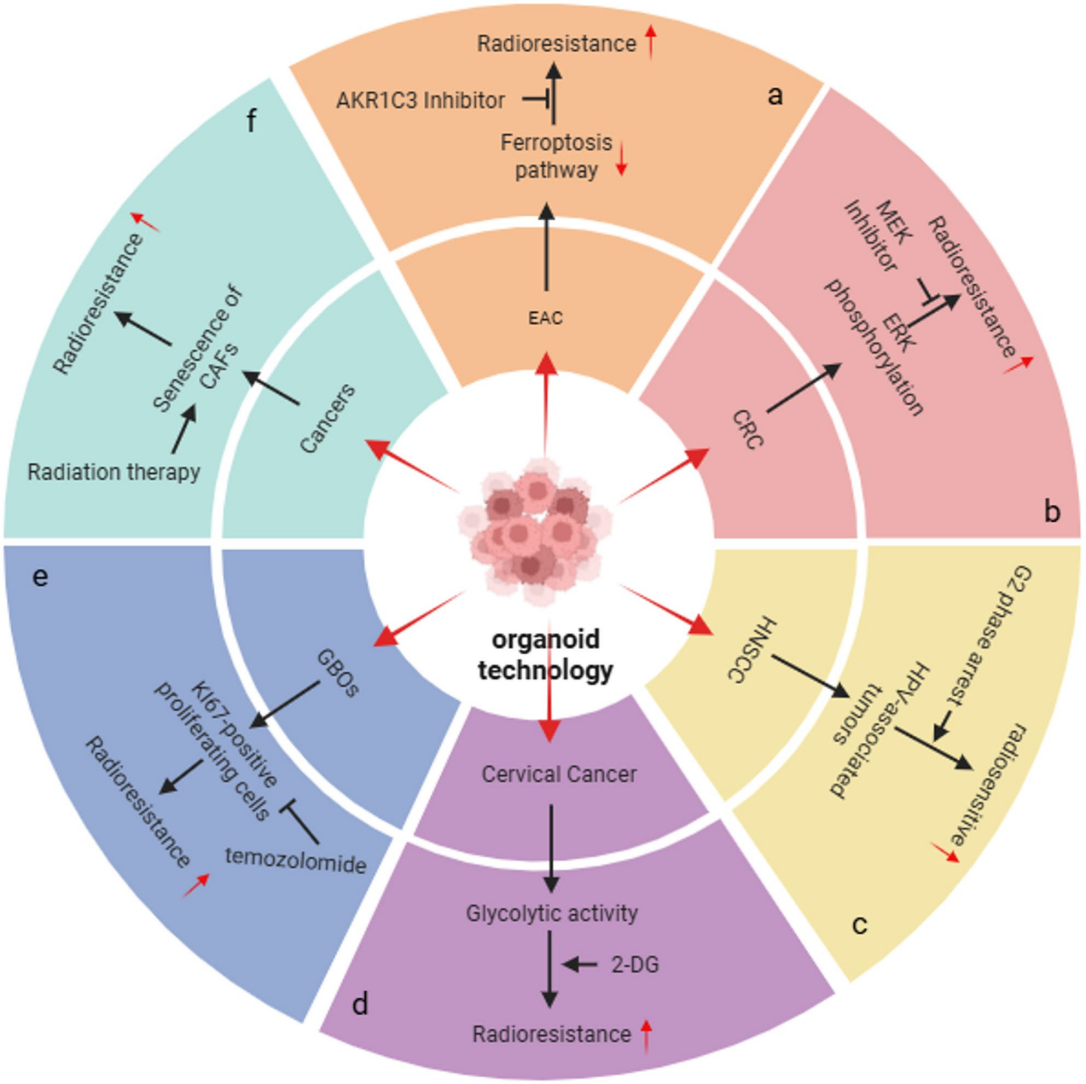
Different tumour organoids. **a** Esophageal adenocarcinoma. **b** Colorectal cancer. **c** Head and neck squamous cell carcinoma. **d** Cervical cancer. **e** Glioblastoma. **f** Multiple cancer types

In gastrointestinal cancer research, organoid models are widely used to elucidate molecular pathways and regulatory mechanisms of radiation response. For instance, studies using esophageal adenocarcinoma (EAC)-derived organoids demonstrated that high expression of AKR1C3 significantly enhances radioresistance by inhibiting the ferroptosis pathway, while its inhibitor can effectively restore radiosensitivity [26] (Fig. 3a). In colorectal cancer (CRC), the organoid platform revealed that the chloride channel inhibitor CaCCinh-A01, by modulating the ANO1/NKCC1 axis and chloride ion homeostasis, achieves radioprotection of normal tissues and radiosensitization of tumors [31] (Fig. 3b). Furthermore, high-throughput drug screening identified MEK inhibitors as radiosensitizers, a mechanism involving the suppression of radiation-induced ERK phosphorylation and RAD51-mediated homologous recombination repair [43]. Quantitative radiosensitivity analysis showed that the D0 values of CRC organoids reflect the dynamic changes in radiosensitivity during tumor malignant progression. Particularly in homologous recombination deficiency subtypes, the PARP inhibitor Olaparib significantly enhanced the radiotherapy response [41]. These studies elucidate the heterogeneity of radiotherapeutic responses in gastrointestinal cancers from multiple perspectives—metabolic regulation, ion homeostasis, DNA damage repair—providing a theoretical basis for targeted combination radiotherapy.

Beyond the aforementioned tumor types, organoid technology also demonstrates precise simulation of molecular subtype-specific radiation responses in head and neck squamous cell carcinoma (HNSCC) and cervical cancer. For example, HNSCC organoids recapitulate the differences in radiosensitivity associated with TP53 mutation and HPV status, confirming that HPV-positive tumors exhibit enhanced radiation response via G2/M phase arrest [52] (Fig. 3c). In cervical cancer organoids, the mesenchymal subtype showed an association between elevated glycolytic activity and radioresistance, and the glycolytic inhibitor 2-DG could reverse this resistant phenotype and induce mesenchymal-to-epithelial transition [70] (Fig. 3d). These results indicate that organoids can effectively link molecular characteristics with functional phenotypes, providing experimental support for biomarker-based stratification of radiotherapy strategies.

In the context of nervous system tumors, patient-derived glioblastoma organoids (GBOs) exhibited heterogeneous patient-specific responses to the combined application of radiotherapy (10 Gy) and temozolomide, with a significant reduction in KI67 + proliferating cells observed in some samples [24] (Fig. 3e). By constructing a co-culture system of mature human brain organoids (MBOs) with glioma cells, the technology modeled tumor-neural microenvironment interactions. It demonstrated that fractionated radiotherapy combined with lncGRS-1 targeted intervention significantly inhibited tumor growth while protecting the viability of normal brain cells [68]. Although these organoid models are not yet perfect in terms of vascular networks and immune components, their 3D structure provides a unique perspective for studying local invasion characteristics and treatment responses.

Expanding the application dimensions further, organoids show significant value in studying tumor microenvironment (TME) regulation. For instance, a co-culture system of organoids with cancer-associated fibroblasts (CAFs) revealed that radiotherapy induces IL-1α-driven senescence in inflammatory CAFs, which in turn promotes radioresistance through stromal remodeling and CD8 + T cell exclusion [48] (Fig. 3f). Targeting IL-1 signaling or using senolytic drugs effectively reversed this process. This study not only highlights the critical role of stromal cells in radiotherapy response but also offers new ideas for microenvironment-targeted combination radiotherapy.

At a deeper mechanistic level, the organoid platform has been utilized to uncover radiation-associated cell death programs and stem cell dynamics. Research indicates that radiation can trigger ferroptosis in intestinal stem cells via the STAT1-IRF1-ACSL4 axis, a process further modulated by arachidonic acid metabolism [57]. Additionally, single-cell transcriptome analysis confirmed that radiation induces the expansion of an Lgr5lo stem cell subpopulation, suggesting stem cell heterogeneity as an important source of radioresistance [28]. These findings deepen the understanding of radiation damage repair and regeneration mechanisms and provide new directions for targeting resistant stem cell subpopulations.

In summary, organoid technology, by highly simulating the genetic background, molecular subtypes, microenvironment composition, and cellular heterogeneity of different tumors, systematically reveals multiple mechanisms influencing radiotherapy response, including metabolic adaptation, DNA repair efficiency, stem cell plasticity, and stromal signaling [72]. This platform not only provides a reliable tool for radiobiological mechanism research but also offers strong support for the development and clinical translation of personalized radiotherapy strategies. With further advancements in co-culture systems, multi-omics integration, and engineered microenvironments, organoid models are poised to play an even more critical role in the field of precision radiotherapy research [42, 73, 74] (Fig. 3).

### Organoid technology combined with multi-omics technology to address the impact of radiation therapy on tumours

The heterogeneity of tumor resistance to radiotherapy represents a major obstacle to achieving precision radiotherapy in clinical practice, stemming from the complex intrinsic molecular networks and dynamically evolving microenvironment of tumors [75]. Systematically deciphering this challenge necessitates a novel research paradigm capable of simultaneously recapitulating patient-specific tumor characteristics and providing multi-layered molecular readouts [76]. In recent years, organoid technology has advanced rapidly due to its ability to highly simulate the tissue architecture, cellular heterogeneity, and key pathophysiological features of patient tumors in vitro. When deeply integrated with multi-omics analysis technologies, it forms a powerful research system: the organoid platform provides highly biomimetic biological samples for multi-omics, while multi-omics technologies endow the organoid models with systematic molecular interpretative depth. This integration enables closed-loop research from clinical phenotypes to molecular mechanisms, greatly advancing the elucidation of radioresistance mechanisms and the development of personalized treatment strategies [77–81].

The starting point of this integrated paradigm lies in establishing the link between genetic background and radiotherapy response. Organoid models can stably maintain the gene mutation spectra and copy number variations of the primary tumor, providing a genetically authentic experimental vehicle for linking genetic alterations to radiosensitivity phenotypes. For instance, in colorectal cancer (CRC) organoids, whole-exome sequencing confirmed their high retention of primary tumor driver mutations, such as BRAF V600E, enabling the analysis of its association with radioresistant phenotypes, such as higher D0 values and enhanced DNA repair activity [25]. Similarly, studies utilizing head and neck cancer organoid biobanks, by integrating genomic sequencing with radiation response data, identified TP53 mutation status and NF-κB pathway activation as molecular markers predictive of radiotherapy tolerance [52]. These organoid-based genomic studies not only provide an initial genetic explanation for radiosensitivity differences but, more importantly, lay a solid molecular foundation for subsequent in-depth functional mechanistic exploration at the transcriptomic and proteomic levels.

Building upon the precise genetic background, transcriptomic analysis further reveals the dynamic regulatory networks of radiotherapy response, translating static genetic sequence information into dynamic gene expression profiles. For example, RNA sequencing of esophageal adenocarcinoma (EAC) organoids identified AKR1C3 as a key upregulated gene associated with radioresistance [26]. In-depth analysis of CRC organoids revealed that radioresistant models significantly upregulate antioxidant metabolism and DNA repair pathways both at baseline and post-irradiation, and CRISPR-Cas9 technology was used to validate the causal role of key genes like GCLC [75]. Single-cell RNA sequencing (scRNA-seq) pushes the research to single-cell resolution, such as identifying the expansion of an Lgr5lo stem cell subpopulation after radiation in intestinal organoids or revealing radiotherapy-induced cell state transitions in medulloblastoma organoids [28, 82]. These transcriptomic findings not only explain how genomic variations influence gene expression to ultimately produce phenotypic outputs but, crucially, they provide clear candidate targets and pathway directions for deeper mechanistic validation at the level of protein function and metabolic activity.

The molecular changes suggested by transcriptomics require proteomics to provide direct functional evidence at the level of post-translational modifications and protein interactions, thereby completing the mechanistic loop from “expression” to “function.” For instance, proteomic analysis confirmed the transcriptomic finding that AKR1C3, by binding to the chaperone HSPA5, inhibits the ubiquitination degradation of GPX4, thus elucidating at a molecular detail how it suppresses radiotherapy-induced ferroptosis [26]. Phosphoproteomics identified the critical role of AMPK phosphorylation in regulating ferroptosis during radiation-induced intestinal injury [57]. Furthermore, cutting-edge technologies like SUMOylation proteomics further reveal epigenetic regulatory mechanisms, such as how SENP5 deficiency enhances the SUMOylation of nuclear proteins to suppress homologous recombination repair efficiency [65]. This protein-level evidence corroborates and builds upon transcriptomic findings, significantly enhancing the completeness and reliability of the mechanistic explanation.

Radiotherapy, as a potent cellular stressor, induces significant metabolic reprogramming. The integration of metabolomics with organoid technology directly reveals the central role of this metabolic adaptability in radioresistance. Studies using high-resolution mass spectrometry for untargeted metabolomic analysis of organoids found that serine/glycine restriction leads to suppressed TCA cycle metabolites in radioresistant organoids, and pyruvate supplementation could reverse the radioresistant phenotype, pointing to the potential of metabolic intervention as a sensitization strategy [83]. Optical metabolic imaging can even quantify metabolic heterogeneity at the single-cell level, identifying metabolically resistant subpopulations difficult to detect by traditional methods [46]. The dynamic changes in metabolites revealed by metabolomics not only correlate with the activity changes of metabolic pathways indicated by transcriptomics and proteomics but also, from the ultimate level of cellular function execution, explain how cancer cells adapt their intrinsic metabolic state to survive radiation stress.

Elevating the research perspective from population averages to single-cell resolution within a three-dimensional space is key to understanding tumor heterogeneity and microenvironment interactions. The combination of spatial multi-omics and single-cell technologies with organoid models successfully achieves this leap. For example, spatial transcriptomics in glioblastoma organoids (GBOs) precisely localized ALDH1A3⁺ glioma stem cells enriched in the hypoxic core, which highly expressed resistance genes, revealing how the spatial microenvironment shapes radioresistance [38]. Single-cell multi-omics analysis can simultaneously resolve transcriptomic and epigenomic information from the same cell, discovering that resistant stem cell subpopulations after radiotherapy not only exhibit activation of Notch/Wnt pathways but also increased openness of H3K27ac-modified regions, elucidating coordinated transcriptional and epigenetic regulatory mechanisms [40]. Such research anchors genomic, transcriptomic, proteomic, and metabolomic information to specific cell subpopulations and spatial locations, thereby mapping the cellular origins and microenvironmental drivers of radioresistance with unprecedented precision.

Ultimately, the value of the organoid-multi-omics integrated platform is reflected in its strong potential for clinical translation. This platform, through systematic data integration, significantly facilitates biomarker discovery and the construction of personalized treatment strategies. For instance, a radiosensitivity gene signature constructed based on multi-omics data was effectively validated in a large number of patient-derived organoids [66]. More importantly, the organoid platform supports high-throughput drug screening, making it possible to “test” drugs for patients in vitro, greatly accelerating the translation of radiosensitizers like PARP inhibitors from mechanistic research to exploration of clinical combination regimens [27, 43]. Several combination strategies discovered based on this platform have entered clinical trial stages, marking the successful translation of this research paradigm from basic findings into precision medicine practices that hold promise for improving patient outcomes.

In summary, the deep integration of organoid technology with multi-omics analysis constructs a systematic, multi-layered research paradigm spanning genetic background, transcriptional regulation, protein function, metabolic remodeling, and spatial heterogeneity, progressively revealing the complex molecular network of tumor radiotherapy response. This strategy not only achieves an in-depth decoding of radioresistance mechanisms from macroscopic phenotypes to microscopic causes but, more importantly, provides powerful technical support and a translational pathway for developing predictive biomarkers and personalized radiotherapy sensitization strategies. It is currently driving, and will continue to propel, tumor radiotherapy into a new era of precision medicine [84, 85].

### Organoid technology guides personalised radiotherapy for tumours

Tumor radiotherapy is undergoing a critical transition from traditional “one-size-fits-all” regimens towards “individually tailored” approaches. In this paradigm shift, organoid technology, leveraging its unique capacity to highly simulate patient-specific tumor biology, provides a powerful platform for advancing personalized radiotherapy. By constructing patient-derived three-dimensional in vitro models, researchers can systematically assess tumor radiosensitivity prior to treatment, offering crucial evidence for clinical decision-making [17, 61, 86, 87]. For instance, in head and neck cancer studies, the in vitro radiosensitivity of organoids significantly correlated with patient recurrence-free survival (log-rank test *p* = 0.01) [42]. Similarly, in glioblastoma models, organoids characterized by molecular markers such as IDH, TERT, and MGMT status accurately predicted patient response to temozolomide combined with radiotherapy (*p* > 0.05 for MGMT unmethylated cases) [38]. These findings establish the significant value of organoids as predictive tools for radiotherapy sensitivity.

Building upon accurate prediction, the organoid platform further serves as an “in vitro testing ground” for customizing individualized radiosensitization strategies. For patients predicted to be radioresistant, the high-throughput drug screening capability of organoids can rapidly identify effective combination therapies. Studies demonstrate that for MGMT unmethylated glioblastoma organoids, combining the PARP inhibitor Veliparib with radiotherapy prolonged the retention of the DNA damage marker γH2AX by three-fold (*p* < 0.001) and increased model survival by 50% [38]. Notably, organoids can also model the complexity of the tumor microenvironment; for example, co-culture with cancer-associated fibroblasts validated the role of the TGF-βR inhibitor Galunisertib in reversing stroma-mediated radioresistance (70% reduction in metastasis rate, *p* < 0.001) [48]. This research pathway—from prediction to intervention—highlights the systematic value of organoid technology in guiding personalized treatment decisions.

Beyond enhancing tumor kill, organoid technology also plays a vital role in balancing efficacy and safety. By establishing paired cultures of tumor organoids and healthy organoids from the same patient, researchers can simultaneously evaluate the therapeutic benefit and potential toxicity of radiotherapy regimens. Research found that the compound I3A specifically promoted crypt repair in normal intestinal organoids without exerting a protective effect on colorectal cancer organoids [67]. In salivary gland models, the mitophagy inducer Urolithin A alleviated radiation damage and promoted stem cell functional recovery [54]. This “tumor-normal tissue” paired testing system provides a unique perspective for optimizing the therapeutic window and achieving individualized dose selection.

Ultimately, the clinical value of organoid technology is fully realized through its ability to accelerate translational research. Technological advances, such as microfluidics, have shortened the organoid drug screening cycle to 7 days, with costs contained under USD 2000 per case, providing a feasible time window for clinical decision-making [66]. More importantly, personalized treatment plans based on organoid predictions have demonstrated application potential in prospective clinical trials. For instance, IDH-mutant glioblastoma patients, guided by organoid testing to enroll in trials combining targeted drugs with radiotherapy, showed a significantly improved objective response rate of 45% (compared to 20% in non-screened groups, *p* < 0.05) [38]. These advances signify the steady progression of organoid technology from a basic research tool towards clinical application.

In summary, organoid technology, by integrating functional prediction, mechanistic dissection, and clinical validation, constructs a comprehensive guidance system for personalized radiotherapy. This system encompasses not only efficacy optimization and toxicity management but also accelerates the translation of research findings into clinical practice [10, 72]. With continued advancements in model standardization and microenvironment simulation technologies, organoid technology holds the promise of truly ushering tumor radiotherapy into a new era of highly personalized “one-size-fits-one” precision medicine.

### Application of organoid-based xenograft technology in tumor radiotherapy research

Organoid-based xenograft technology has emerged as a significant tool in recent cancer research, demonstrating considerable potential in the field of radiotherapy studies. By transplanting patient-derived organoids into immunodeficient animals, this technology establishes preclinical models that closely mimic human tumor biology, providing a reliable platform for investigating radiosensitivity mechanisms and evaluating combination therapy strategies. The specific applications of this technology are systematically elaborated below, focusing on model characteristics, personalized predictive capability, mechanistic research depth, and clinical translation value.

Regarding model establishment, organoid-based xenograft technology highly preserves the histological architecture, genetic background, and heterogeneity of the primary tumor, thereby effectively simulating clinical radiotherapy responses. For instance, in rectal cancer research, transplantation of patient-derived organoids into the mouse rectal mucosal layer, combined with precise local irradiation using small animal irradiation platforms, successfully recapitulated patient-specific radioresistance phenotypes, including key features such as cancer-associated fibroblast enrichment and T-cell exclusion [48]. In glioblastoma studies, intracranial transplantation of organoids modeled the tumor’s invasive growth and its heterogeneous response to radiochemotherapy, highlighting the technology’s advantage in maintaining the in situ characteristics of tumors [24].

In the realm of personalized therapy prediction, organoid-based xenograft models demonstrate high concordance with patient clinical outcomes. Multiple studies, utilizing in vitro organoid radiosensitivity testing coupled with xenograft validation, have confirmed their effectiveness in predicting radiotherapy response. For example, in head and neck squamous cell carcinoma, the differential radiosensitivity of organoids significantly correlated with patient recurrence-free surviva [88]. In cervical cancer organoid models, low expression of NSUN6 was associated with increased radiosensitivity in organoids, and correspondingly, with better patient prognosis, underscoring the platform’s practical value in individualized outcome prediction [89].

At the mechanistic exploration level, organoid-based xenograft models enable in-depth dissection of radioresistance mechanisms within the tumor microenvironment. Researchers can employ genetic editing tools to specifically modulate target genes in organoids, followed by validation of their role in radiation response through in vitro and in vivo experiments. For instance, in colorectal cancer models, knockdown of SENP5 enhanced radiation-induced DNA damage and suppressed tumor growth, effects verified in both organoid and xenograft settings [65]. Other studies using this model have revealed the central role of the IL-1/iCAF axis in mediating rectal cancer radioresistance, providing an experimental basis for targeted interventions [48].

Despite the prominent advantages of organoid-based xenograft technology in radiotherapy research, it faces limitations concerning model completeness and technical accessibility. These models often lack a fully functional immune microenvironment and vascular structures, which may impair the evaluation of immunomodulatory therapies or hypoxia-related radiation responses. Furthermore, their widespread adoption is hindered by prolonged model establishment timelines, technical complexity, and substantial costs [24]. For example, glioblastoma organoids tend to lose native microenvironmental cells during long-term culture, and establishing rectal cancer organoid xenograft models relies on specialized equipment and animal facilities, hindering widespread adoption [43, 48].

In conclusion, organoid-based xenograft technology, by faithfully recapitulating patient-specific tumor characteristics, enabling personalized radiotherapy prediction, and supporting profound mechanistic investigation, has become an indispensable tool in radiotherapy research. Future efforts integrating humanized immune systems, optimizing 3D culture systems, and conducting multi-center, large-sample validation are expected to further advance the personalization and precision of tumor radiotherapy.

### Preclinical and technical limitations of organoid technology

Although patient-derived organoid (PDO) technology serves as a promising bridge between basic research and clinical practice, particularly in modeling patient-specific tumor biology and treatment responses, its translation from an experimental platform to routine clinical application is hindered by a series of preclinical and technical bottlenecks. These limitations, stemming from inherent model system simplifications and operational workflow constraints, collectively compromise its predictive accuracy, reproducibility, and feasibility for clinical integration.

A primary challenge is the limited ability of current organoid models to recapitulate the full complexity of the native tumor microenvironment (TME). Standard culture systems predominantly rely on basement membrane extracts (e.g., Matrigel) as a 3D scaffold, which, while supporting epithelial tumor cell growth, actively excludes critical TME components such as immune cells, vascular networks, cancer-associated fibroblasts (CAFs), and innervation [26, 27]. This absence of key microenvironmental elements, notably highlighted in studies of esophageal adenocarcinoma, colorectal, and head and neck cancers, prevents the model from replicating vital biological processes including immune regulation, cytokine networks, and stroma-epithelial interactions. Consequently, this simplification may lead to an overestimation of the efficacy of targeted drugs or radiosensitizers and restricts the evaluation of combination immunotherapies [24, 30, 31, 52]. The incomplete TME directly results in insufficient physiological relevance, which in turn underscores the urgent need for technical standardization to enhance the model’s predictive reliability.

Secondly, significant challenges in standardization and reproducibility adversely affect the reliability of results across different laboratories and multi-center clinical applications. Organoid culture success rates exhibit considerable variability, ranging from 76.2% for meningiomas to as low as 17% in some studies, and are highly dependent on sample quality, processing timeliness, and patient treatment history [37, 58]. Batch-to-batch variations and inherent individual heterogeneity in organoid size, cellular composition, and metabolic state, coupled with potential genetic drift or loss of critical tumor subpopulations (e.g., TP53 mutant clones) during long-term passaging, undermine model stability and clinical representativeness [30, 52]. Furthermore, difficulties in optimizing medium composition, batch variability of Matrigel, and a lack of standardization in functional assay platforms (e.g., drug screening Z-factor values fluctuating between 0.3 and 0.92) exacerbate result variability. This creates uncertainty in translating PDO-based drug sensitivity data into predictable clinical efficacy [39, 88].

Moreover, the clinical predictive value of organoid models is constrained by a fundamental mismatch between the simplified in vitro system and the complex in vivo physiological environment. Current models struggle to simulate systemic drug pharmacokinetics, tissue-specific toxicities, or long-term dynamic processes post-treatment, such as radiation-induced fibrosis or abscopal immune effects [27, 41, 50]. For instance, PDOs failed to predict the dose-limiting toxicity of CFI-400,945 observed in clinical trials and cannot fully capture the dynamic evolution of the TME or immune editing processes following radiotherapy [26, 57]. Although clonogenic assays using organoids can reflect cell-autonomous radiosensitivity, their inadequate modeling of microenvironment-mediated resistance mechanisms and intra-tumoral genetic heterogeneity limits their direct utility in optimizing personalized radiotherapy regimens [58]. This lack of physiological relevance not only impairs accurate treatment response prediction but also exposes inherent limitations in simulating systemic physiological barriers and dynamic processes.

Finally, the practical integration of PDO technology into clinical workflows faces real-world barriers related to unstable success rates, insufficient timeliness, and low cost-effectiveness. The success rate of generating PDOs varies significantly across cancer types, with rates for tumor tissue (28.4%) being substantially lower than for adjacent normal tissue, and rates for metastatic samples can be as low as 4.5% [24, 42]. The typical 3–4 week timeline required for organoid establishment and subsequent drug sensitivity testing conflicts with the urgent 1–2 week window for clinical decision-making. While microfluidic technologies can potentially shorten this cycle to 7 days, the high per-sample cost (approximately 2,500 − 5,000) and lack of insurance coverage severely limit clinical accessibility [66, 90]. These factors collectively highlight the technical bottlenecks in standardization, automation, and cost-effectiveness optimization that must be overcome for the technology to move from the laboratory to large-scale clinical application.

In conclusion, while organoid technology holds significant promise for personalized oncology research, its clinical application is currently constrained by multiple preclinical and technical limitations, including inadequate TME simulation, lack of standardization, limited physiological relevance, and low construction efficiency. Future advancements require the development of immunocompetent/vascularized co-culture systems, establishment of automated and standardized protocols, integration of dynamic monitoring technologies, and the execution of large-scale prospective clinical trials. These efforts are essential to systematically enhance the biological completeness, data reliability, and clinical translational value of PDO models, thereby facilitating their substantive transition from a basic research tool to a clinically impactful application [26, 27, 30, 37, 42, 46, 48, 52, 58, 88].

### Organoid technology possesses significant potential for clinical application

Organoid technology, with its capacity to model patient-specific 3D microenvironments with high fidelity (> 85%), is systematically reshaping clinical pathways in oncology. In personalized treatment decision-making, glioblastoma organoids (GBOs) accurately predict therapeutic outcomes by integrating molecular profiling (IDH/TERT/MGMT) [91]. For instance, MGMT-unmethylated patients showing resistance to temozolomide combined with radiotherapy can be redirected to a PARP inhibitor (Veliparib) sensitization strategy, boosting survival rates by 50% [92]. Furthermore, this “patient avatar” model facilitates rapid drug screening, identifying salvage therapeutics like Costunolide within three weeks to extend survival in recurrent cases [38]. This platform is revolutionizing drug discovery—single-cell sequencing has pinpointed ALDH1A3⁺ therapy-resistant stem cell subpopulations within hypoxic niches of GBOs, accelerating the development of the IDH1 inhibitor Ivosidenib and enabling its precise enrollment in Phase II trials combined with radiotherapy, thereby increasing the objective response rate from 20% to 45% [38]. In head and neck cancer research, a 5-gene radiosensitivity signature (involving CDK1/XRCC1, etc.) derived from organoids enables high-precision prediction, guiding dose de-escalation to 35 Gy to reduce mucosal toxicity. The integration of microfluidic platforms (e.g., MO platform) further compresses the testing timeline to just 7 days, effectively resolving delays associated with traditional workflows [21, 59].

Organoid technology also serves as a core accelerator for optimizing combination therapies. Spatial transcriptomics has revealed that tertiary lymphoid structure (TLS) germinal center B-cell enrichment (a 2.1-fold increase) serves as a biomarker for benefit from anti-PD-1 therapy combined with radiotherapy in head and neck cancer (45% ORR improvement). In ovarian cancer, targeting the ET-1/YAP pathway with Atrasentan resulted in a 70% reduction in metastasis rates post-combination therapy, demonstrating the transformative value of microenvironmental dissection for rational combination design [59, 93]. Regarding clinical translation, automated GMP-compliant systems aim to reduce costs to $1000 per case by 2026. Prospective clinical trials (e.g., NCT04833170) are validating survival benefits, providing a clear pathway for implementing novel strategies like LOX inhibitors combined with SBRT [66, 94]. Reimbursement initiatives (targeting 30% co-pay by 2026 under DRG schemes) and the establishment of international databases (e.g., ICORG, targeting ISO 20387 accreditation by 2025) are creating the necessary payment and regulatory frameworks for widespread clinical adoption [48]. Notably, organoid platforms address critical gaps in rare tumor research—salivary gland cancer models preserving native ductal structures guide immunotherapy screening for sarcomas (reducing prediction bias by 30%), filling a void left by traditional models [21].

With advancements in bioprinting vascular networks to better simulate hypoxia (recreating 0.5%−21% oxygen gradients) and co-culture systems incorporating T cells and CAFs to enhance immune interaction modeling, organoid technology is evolving from a mere predictive tool into an “editable microenvironment decision engine.” By integrating anatomical fidelity (e.g., blood-brain barrier, pelvic mechanics), functional high-throughput screening (7-day drug sensitivity), and dynamic molecular tracking (epigenetic profiling), it empowers a fundamental shift from “one-size-fits-all” regimens towards a “one-patient-one-protocol” paradigm. The ultimate goal is its mandatory inclusion in NCCN guidelines by 2026, aiming to drive a 50% improvement in 5-year survival rates—a true clinical transformation powered by this disruptive technology [95].

## Conclusion

Organoid technology, as a revolutionary three-dimensional in vitro model, is fundamentally transforming our understanding of the complex mechanisms by which the tumor microenvironment (TME) influences response to radiotherapy. This review systematically elaborates on the significant advantages of organoids in simulating TME heterogeneity, cell-cell interactions, immune components, stromal architecture, and hypoxic conditions, thereby uncovering the profound regulatory roles these elements play in radiotherapy outcomes. Research demonstrates that organoids not only highly retain the genetic features and histological diversity of patient tumors but also functionally recapitulate key TME-mediated pathways of radioresistance, providing a powerful platform that overcomes the clinical relevance limitations of traditional models.

In the realm of personalized radiotherapy, organoid models demonstrate considerable translational value. The establishment of patient-derived organoid (PDO) biobanks enables high-throughput in vitro screening of radiosensitizing drugs, optimization of radiation dosing regimens, and prediction of inter-individual response variations. Furthermore, the integration of organoids with multi-omics technologies (e.g., genomics, transcriptomics, proteomics) and engineering approaches like microfluidic chips enhances their capacity to decipher the molecular mechanisms underlying radioresistance, offering novel insights for developing combination strategies such as radio-immunotherapy and radio-targeted therapy. Notably, organoids have been validated in radiosensitivity studies across various cancers, including colorectal, pancreatic, and breast cancer, with their predictive consistency for clinical responses providing preliminary evidence of their reliability as a biomarker platform.

However, the widespread clinical application of organoid technology still faces challenges. Current models have limitations in simulating a complete TME, including vascular systems, innervation, and systemic immune context. The lack of standardized culture protocols, the mismatch between culture timelines and clinical decision-making windows, and the cost-effectiveness of building large-scale biobanks represent significant technical bottlenecks requiring urgent solutions. Future research should focus on developing more complex models, such as immunocompetent and vascularized organoids, combined with artificial intelligence-assisted high-throughput functional analysis to improve their fidelity in mimicking in vivo physiology. Concurrently, promoting organoid-guided prospective clinical trials will be crucial for validating their efficacy in personalizing radiotherapy and establishing their standardized role in precision oncology.

In summary, organoid technology, by precisely modeling the TME, provides an unprecedented tool for elucidating radioresistance mechanisms and optimizing therapeutic strategies. Despite current limitations, the continued integration of engineering technologies and molecular biology holds the promise that organoid models will become a critical bridge connecting basic research and clinical practice in the near future, ultimately steering tumor radiotherapy towards greater efficacy and personalization.

## Data Availability

Not applicable.
